# Editorial: NK cells in transplantation

**DOI:** 10.3389/fimmu.2026.1882415

**Published:** 2026-05-28

**Authors:** Shannon R. McCurdy, Hiba Azar, Asma Beldi-Ferchiou

**Affiliations:** 1 Division of Hematology and Oncology Disease, Department of Medicine, Hospital of the University of Pennsylvania, Philadelphia, PA, United States; 2 Division of Nephrology, Department of Medicine, Hotel Dieu de France University Hospital, Beirut, Lebanon; 3 Biological Hematology and Immunology department, APHP, Hôpital Henri Mondor, Créteil, France; 4 Univ Paris Est Créteil, INSERM U955, Institut Mondor, Créteil, France

**Keywords:** NK cells, organ transplantation, post-transplant complications, post-transplant reconstitution, prognosis factors

Natural killer (NK) cells are innate lymphoid cells with a well-established role in virus and tumor surveillance. They express a large repertoire of receptors, which can recognize and eliminate abnormal cells without prior sensitization (1). The interplay of signals transmitted by both their activating and inhibitory receptors tightly regulates NK-cell behavior (1). Beyond their capacity to “kill”, NK cells also secrete a variety of proinflammatory cytokines and interact with other immune cells to shape immune response. The efficacy of many drugs (e.g., Rituximab) largely depends on NK cell–mediated antibody-dependent cellular cytotoxicity. In the context of transplantation, NK cells have gained increasing attention due to their rapid post-graft reconstitution, their involvement in post-transplant viral immunity, and their graft-versus-leukemia effects. However, NK cells can also negatively regulate T-cell responses to viral and allo-antigens (2). For instance, by killing allogeneic antigen presenting cells, NK cells may promote graft tolerance and decrease rates of graft-versus-host disease (GVHD) (3). In contrast, “missing self” class I MHC molecules can induce NK-cell activation and trigger chronic rejection in kidney transplantation (KT) (4).

The intricate balance between activating and inhibiting receptors and the role of NK cells in both graft tolerance and graft rejection highlights the complexity of NK-cell biology and the need for further research (5). This Research Topic *“Natural Killer Cells in Transplantation”* is a Research Topic of original articles that contribute to our knowledge of the role of NK cells in transplantation. Two fields in which NK cells are of prime interest are allogeneic hematopoietic stem cell transplantation (HSCT) and kidney transplantation. In keeping, this topic includes three articles investigating the role of NK cells in HSCT and one article exploring NK cells in KT. We highlight a few of their important findings below.

Pesˇic et al. focused on immune reconstitution after HSCT and showed that higher NK-cell counts at day 90 after allogenic transplantation with antithymocyte globulin (ATG) T cell depletion are independently associated with improved overall survival (OS) and reduced non-relapse mortality (NRM). The authors posited that the reduction in NRM was due to fewer viral, particularly cytomegalovirus-related deaths in patients with higher NK-cell counts. These data suggest that NK-cell reconstitution is a biomarker for clinical outcomes, particularly in settings in which T-cell numbers are initially reduced by GVHD prophylaxis.

Not only are NK cells vital to the outcomes in allo-immune settings, but they also impact outcomes in autologous HSCT. Astarloa-Pando et al. demonstrated that early after auto-HSCT, NK cells transiently acquired a decidual-like phenotype and exhibit an immature phenotype with increased proportion of CD56^bright^ NK cells and an enrichment of the less differentiated NKG2A+ CD57- fraction within CD56^dim^ subset. In parallel, NK cells acquire an activated state along with an upregulation of inhibitory receptors and a downregulation of activating ones. Data obtained on cytokine profiles suggest that IL-15 may contribute to the activation phenotype of NK cells. These findings indicate that rather than a simple quantitative recovery, NK-cell reconstitution involves profound qualitative changes that may influence their functional capacity. Importantly, authors report that in non-Hodgkin lymphoma patients, lower frequencies of immature NKG2A+CD57- NK cells or higher frequencies of mature CD57+ NK-cell subset at 30 days post auto-HSCT exhibited significantly improved progression-free survival (PFS).

Similar to autologous HSCT, the phenotype of the recovering NK cells is profoundly altered early after allogeneic HSCT. For instance, in platforms using cyclophosphamide post transplantation (PTCy), after an early ablation of mature dividing NK cells, high levels of IL-15 lead to rapid recovery of immature NK cells expressing CD62L^+^NKG2A^+^KIR^-^ whereas mature NK-cell recovery is delayed (6). Other studies also highlight the immaturity of NK cells early post-HSCT that could be potentially altered by low-dose IL-15 administration (7). Furthermore, higher NK-cell recovery and NK-cell inflammatory signatures at Day 28 post-HSCT were associated with improved OS (8), due to a reduction in relapse in patients receiving PTCy. Thus, both NK-cell differentiation and functional status are key determinants of clinical outcomes. Altogether, this work underscores the importance of considering NK-cell phenotype when evaluating immune recovery.

One area of NK-cell biology that is particularly complex which has differing impact depending on the GVHD prophylaxis strategy is the effects of killer immunoglobulin-like receptors (KIRs) matching and genotype. Tang et al. highlight the importance of NK-cell alloreactivity in a cohort of pediatric patients undergoing haploidentical transplantation with ATG-based GVHD prophylaxis, demonstrating that specific KIR–HLA combinations improved survival. Specifically, there was a significantly reduced risk of relapse when pediatric recipients lacking HLA-A3/A11 received HSCT from donors with both KIR3DL2 and HLA-A3/A11. The absence of recipient HLA ligands (including HLA-A3/A11) for donor inhibitory KIR (KIR3DL2) has been shown to be associated with lower relapse incidence in other studies using T-cell depletion with ATG, supporting the validity of this association (9).

While these findings provide further evidence that NK-cell alloreactivity mediates graft versus leukemia effect, the diversity of KIRs and the balance of inhibition and activating signals makes it difficult to achieve consistent results, particularly in non-T cell-depletion platforms. For instance, when using PTCy-based GVHD prophylaxis, KIR mismatching has been associated with higher relapse, less relapse, and no effect on outcomes, depending on the study (10–14).

The role of NK cells in solid organ transplantation is even less well understood. Beldi-Ferchiou et al. explored the association of pre-KT absolute numbers and percentages of T-, B- and NK-cell populations with opportunistic infections (OI) and acute rejection post-KT. Surprisingly, they found that higher pre-transplant NK-cell counts were associated with an increased risk of OI within two years after KT. These findings were corroborated by another recent report highlighting an association between high NK cells CD56^dim^ frequency before pediatric KT and risk of viral infection after transplantation (4). To explain this unexpected result, they hypothesized that NK and T cells compete for key cytokines (IL-2, IL-15); when T-cell levels are low, cytokines preferentially activate NK cells, limiting T-cell responses. In keeping, higher pre-KT T-cell levels were associated with lower OI rates. In the context of KT, T cells may be highly activated and express stress-induced ligands (e.g., MICA). This could promote their recognition and elimination by NK cells *via* the NKG2D receptor thereby decreasing T cells available to respond to viral or other opportunistic infections (3). These studies emphasize the complex role of NK cells and underscore the importance of integrating NK-cell measurements into immune profiling strategies to better predict post-transplant risks.

In conclusion, this Research Topic highlights the multifaceted role of NK cells that differs according to transplant type, GVHD prophylaxis, and timing of analysis. By integrating clinical observations with detailed immunological analyses, these studies provide a more comprehensive understanding of NK-cell biology. Future research combining immunophenotyping, functional assays, genomic approaches, and machine learning models may help to anticipate and stratify post-transplant risks.
